# Intrafamilial Phenotypic Variability and Dental Management of Ectodermal Dysplasia in Three Siblings: A Case Report

**DOI:** 10.1155/crid/6651113

**Published:** 2026-07-28

**Authors:** Farah Chouchene, Fatma Masmoudi, Ahlem Baaziz

**Affiliations:** ^1^ Dentistry Department Ibn El Jazzar University Hospital, Kairouan Faculty of Dental Medicine of Monastir Laboratory of Biological, Clinical and Dento-Facial Approach, Laboratory of Biological, University of Monastir, Monastir, Tunisia, um.rnu.tn; ^2^ Pediatric and Preventive Dentistry Department, Faculty of Dental Medicine of Monastir, Laboratory of Biological, Clinical and Dento-Facial Approach, University of Monastir, Monastir, Tunisia, um.rnu.tn

**Keywords:** conservative dentistry, ectodermal dysplasia, oligodontia, pediatric dentistry, prosthetic rehabilitation

## Abstract

Ectodermal dysplasia (ED) is a heterogeneous group of inherited disorders affecting ectoderm‐derived structures, with dental anomalies representing a major clinical concern. This case report describes three siblings from a Tunisian family presenting marked intrafamilial phenotypic variability of ED. Clinical and radiographic findings revealed variable patterns of oligodontia (ranging from two to eight missing teeth), peg‐shaped teeth, taurodontism, and craniofacial skeletal discrepancies. Cephalometric analysis demonstrated altered dentoalveolar development, whereas the adolescent patient reported significant psychosocial distress. Management included minimally invasive composite reshaping in two patients and a removable partial denture in the youngest child to restore function and vertical dimension, combined with orthodontic referral and longitudinal growth monitoring over a 2‐year follow‐up period. Despite a shared genetic background, the severity and clinical expression varied considerably, requiring individualized therapeutic strategies. This report emphasizes that early, growth‐adapted, multidisciplinary management is essential in ED and that conservative and interim prosthetic approaches can significantly improve functional, aesthetic, and psychosocial outcomes during childhood.

## 1. Introduction

Ectodermal dysplasia (ED) comprises a heterogeneous group of inherited disorders characterized by abnormal development of two or more structures derived from the embryonic ectoderm, including teeth, hair, skin, nails, and exocrine glands [1–3].

The hypohidrotic form is the most common subtype and is frequently associated with mutations affecting the ectodysplasin signaling pathway. At the molecular level, ED is primarily linked to mutations in the EDA, EDAR, and EDARADD genes, which play a critical role in epithelial–mesenchymal interactions during embryogenesis, particularly in odontogenesis and craniofacial development [4, 5]. Disruption of this signaling pathway leads to abnormalities in tooth number, morphology, and eruption [4, 5].

Dental anomalies represent some of the most consistent and clinically significant manifestations of ED. Common findings include hypodontia or oligodontia, conical or peg‐shaped teeth, delayed eruption, and taurodontism [6–8]. These abnormalities may impair masticatory efficiency, speech development, facial aesthetics, and overall quality of life, especially in pediatric patients [7, 9]. Early tooth agenesis is also associated with reduced alveolar bone development, decreased vertical dimension of occlusion, and altered craniofacial growth patterns, often requiring staged and interdisciplinary management from a young age [8, 10, 11].

Early diagnosis and timely intervention are essential to improve functional outcomes, support harmonious craniofacial development, and enhance psychosocial well‐being [9, 12]. Consequently, conservative restorative approaches and interim prosthetic solutions represent fundamental pillars in the management of pediatric patients [9]. These early rehabilitative strategies allow for the restoration of masticatory efficiency and aesthetics while remaining adaptable to the continuous physiological changes associated with active growth [9, 12].

Despite extensive research on ED, intrafamilial phenotypic variability remains insufficiently explored. Individuals with similar genetic backgrounds may present markedly different clinical manifestations, suggesting variable expressivity and the potential influence of environmental or epigenetic factors [5, 13]. Therefore, the present case report is aimed at describing the clinical and radiographic findings of three siblings affected by ED, highlighting intrafamilial variability and emphasizing the importance of early, multidisciplinary, and growth‐adapted management.

## 2. Case Presentation

This case report adheres to the CARE (CAse REport) guidelines [14]. Written informed consent was obtained from the parents for all procedures and publication of anonymized data.

Family history revealed a nonconsanguineous background, with the mother and all three siblings presenting congenital tooth agenesis, suggesting a hereditary pattern consistent with ED.

The comparative clinical, radiographic, and therapeutic features of the three siblings are presented in Table 1.

**Table 1:** Comparative clinical, radiographic, and therapeutic features of the three siblings.

General features			
Age (years)/sex	5 years/male	8 years/female	13 years/male
Chief complaint	Missing teeth	Screening (familial evaluation)	Aesthetic concern
Extraoral findings	Dry and rough skin	Dry skin	Dry skin
	Sparse hair and eyelashes	Frontal bossing	Sparse hair and eyelashes
	Square forehead	Sparse hair and eyelashes	Frontal bossing
	Frontal bossing.	Depressed nasal bridge.	Depressed nasal bridge
	Depressed nasal bridge		
	Prominent lower lip		
Dental finings			
Number of missing teeth	Six primary teeth	Six permanent teeth (hypodontia/oligodontia)	Two permanent teeth (hypodontia)
	Eight permanent teeth (oligodontia)		
Missing teeth	52, 62, 71, 72, 81, 82, 22, 23, 32, 42, 33, 43, 37, 47	12, 35, 45, 17, 37, 47	35,45
Tooth morphology	Conical incisors	Peg–shaped anterior teeth	Peg–shaped anterior teeth
Other anomalies	—	Ectopic eruption	Taurodontism
		Transposition	
Skeletal pattern			
Radiographic findings	Agenesis + skeletal Class I (Class II tendency)	Ectopic eruption	Agenesis
			Taurodontism
			Class III tendency
Functional/psychosocial impact			
Functional impairment	Mastication/phonation	Mild aesthetic concern	Mainly aesthetic concern
Psychological impact	Minor distress	Minor distress	Significant distress
Management			
Treatment approach	Composite + removable partial denture (RPD)	Interceptive orthodontic	Composite reshaping + orthodontic
Orthodontic treatment	Planned	Interceptive	Completed
Follow–up	Every 6 months for 2 years ongoing follow‐ups	Every 6 months for 2 years completed follow‐up	Every 6 months for 2 years completed follow‐up
Treatment outcomes	Improvement in function and aesthetics	Stable occlusion	Improved aesthetics and confidence

The sequence of therapeutic interventions and follow‐up for each case is illustrated in a timeline diagram (Figure 1).

**Figure 1:**
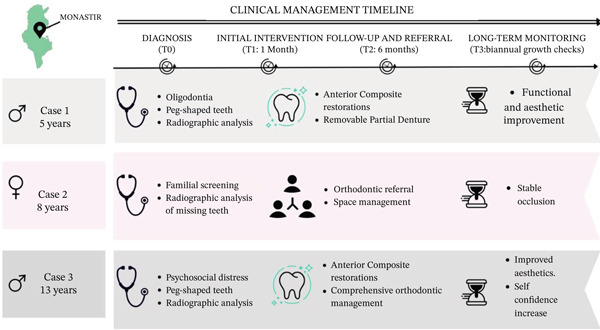
Timeline of clinical management and follow‐up of three siblings.

### 2.1. Treatment Planning and Therapeutic Approach

Management of the three siblings was based on an individualized, growth‐adapted, and multidisciplinary approach. The main therapeutic objectives were to restore or preserve oral function, improve aesthetics and psychosocial well‐being, maintain occlusal stability, and support harmonious craniofacial development. Treatment planning considered the patients′ age, severity of dental anomalies, skeletal relationships, and future rehabilitation needs.

Conservative restorative procedures, removable prosthetic rehabilitation, and interceptive orthodontic strategies were selected as minimally invasive and growth‐compatible approaches. Definitive prosthetic rehabilitation was postponed until completion of craniofacial growth.

### 2.2. Case 1: Youngest Sibling (5‐Year‐Old Boy)

#### 2.2.1. Diagnosis and Etiology

A 5‐year‐old boy was referred to the Department of Pediatric Dentistry at the Clinic of Dental Medicine of Monastir due to congenital absence of multiple teeth. His medical history was unremarkable, with no history of dental trauma or extraction.

Extraoral examination revealed dry, rough skin, sparse eyelashes, and fine, brittle scalp hair.

Facial dysmorphisms included a square forehead, frontal bossing, depressed nasal bridge, and prominent lower lip. Nail morphology was unremarkable.

Intraoral examination demonstrated absence of primary teeth (52, 62, 71, 72, 81, 82), and conical maxillary central incisors (51, 61) (Figure 2).

**Figure 2:**
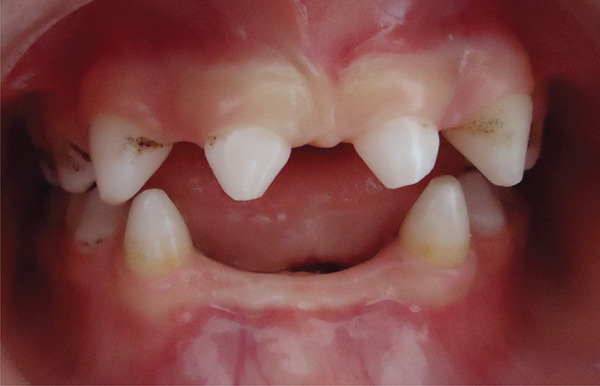
Intraoral photograph revealed the absence of several primary teeth; 52, 62, 71, 72, 81, 82 and the conical shape of incisors 51 and 61.

Panoramic radiography confirmed oligodontia of permanent teeth (22, 23, 32, 42, 33, 43, 37, 47) (Figure 3A). Cephalometric analysis showed skeletal Class I with Class II tendency and mandibular retrognathism (Figure 3B).

**Figure 3:**
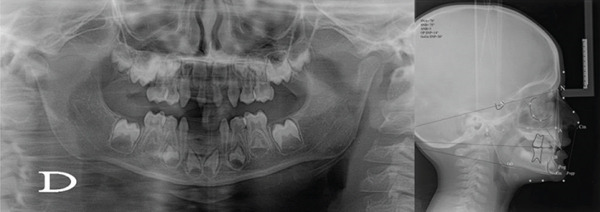
Combined radiographic assessment of Case 1. Panoramic orthopantomogram showing oligodontia of permanent teeth (22, 23, 32, 42, 33, 43, 37, 47). Lateral cephalometric radiograph demonstrating skeletal Class I with Class II tendency and mandibular retrognathism.

#### 2.2.2. Treatment Progress

Treatment consisted initially of direct composite restorations of the maxillary incisors using a nanohybrid composite resin (Filtek Universal Composite, 3 M ESPE, St. Paul, United States; shades A2/B2). A two‐step etch‐and‐rinse adhesive system (Adper Single Bond 2, 3 M ESPE, United States) was applied following selective enamel etching. Composite was placed using an incremental layering technique and finished with Sof‐Lex polishing discs (3 M ESPE). Subsequently, a mandibular removable partial denture (RPD) was fabricated using silicone‐based functional impressions (Zhermack, Italy) and retained with wrought‐wire clasps (Figure 4).

**Figure 4:**
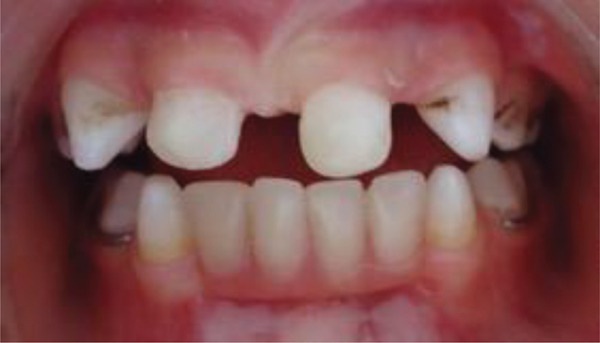
Intraoral photograph showing mandibular denture in occlusion.

#### 2.2.3. Treatment Results

At 6‐months follow‐up, the treatment results showed improved mastication, phonation, and dental aesthetics. The RPD demonstrated satisfactory retention and adaptation.

Follow‐up has been conducted at 6‐month intervals and is ongoing, with periodic adjustments of the prosthesis to accommodate craniofacial growth and maintain functional stability.

### 2.3. Case 2: Elder Sister (8 Years Old)

#### 2.3.1. Diagnosis and Etiology

The elder sister, aged 8 years, was referred to the Department of Pediatric Dentistry for clinical evaluation as part of familial screening.

Extraoral examination revealed phenotypic features consistent with ED including dry skin and sparse eyelashes.

Intraoral findings included absence of Tooth 12, maxillary canine‐premolar transposition (23–24) (Figures 5 and 6), with additional agenesis of mandibular second premolars (35 and 45) and second molars (17, 37, 47) confirmed radiographically (Figure 7).

**Figure 5:**
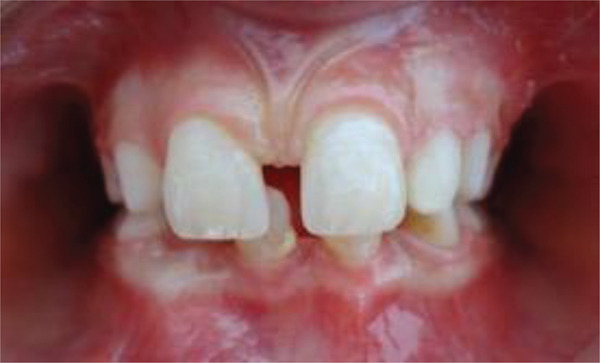
Intraoral frontal view of Case 2.

**Figure 6:**
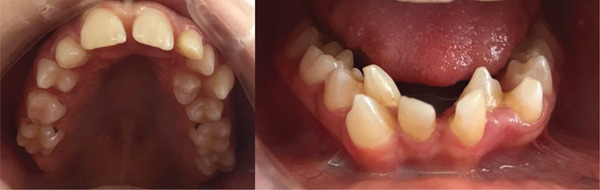
Intraoral view of Case 2 showing maxillary canine‐premolar transposition (23–24) and mandibular mispositioned teeth.

**Figure 7:**
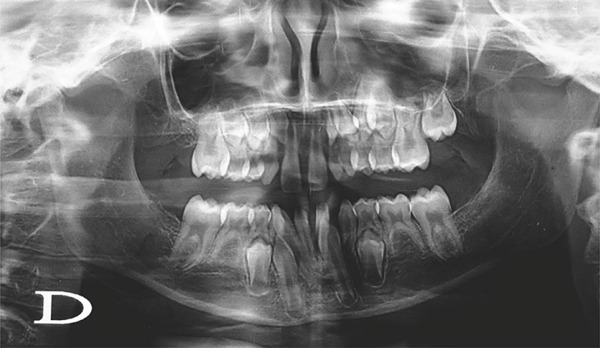
Panoramic radiograph of Case 2 showing congenital absence of Teeth 12 and 32, agenesis of the mandibular second premolars (35 and 45), absence of second permanent molars (17, 37, 47).

#### 2.3.2. Treatment Progress

Management involved referral to the department of orthodontics for growth monitoring and space management planning. Interceptive strategies were initiated to maintain arch integrity and facilitate future rehabilitation.

#### 2.3.3. Treatment Results

At 6‐months follow‐up, treatment results showed stable occlusion and appropriate arch development with no reported complications. Follow‐up was conducted every 6 months over a 2‐year period. Interceptive orthodontic treatment was performed and a prosthetic rehabilitation plan is currently being developed to preserve spaces and allow future implant placement after completion of growth.

### 2.4. Case 3: Older Brother (13 Years Old)

#### 2.4.1. Diagnosis and Etiology

The diagnosis and etiology were based on clinical, radiographic, and psychosocial assessment. The patient presented with aesthetic concerns and reported significant social discomfort.

Extraoral examination revealed sparse hair, frontal bossing, and facial dysmorphisms.

Intraoral findings included peg‐shaped anterior teeth (12, 31, 32, 33, 41, 42, 43), crowding, and malocclusion (Figure 8).

**Figure 8:**
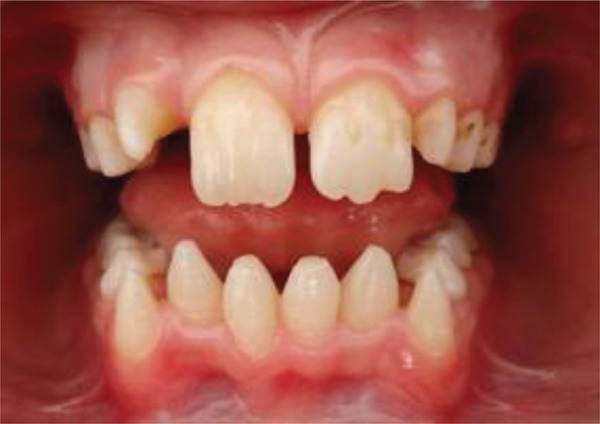
Intraoral photograph showing anterior peg‐shaped teeth.

Radiographic analysis showed agenesis of Teeth 35 and 45, along with taurodontism affecting molars (16, 26, 36, 46) (Figure 9).

**Figure 9:**
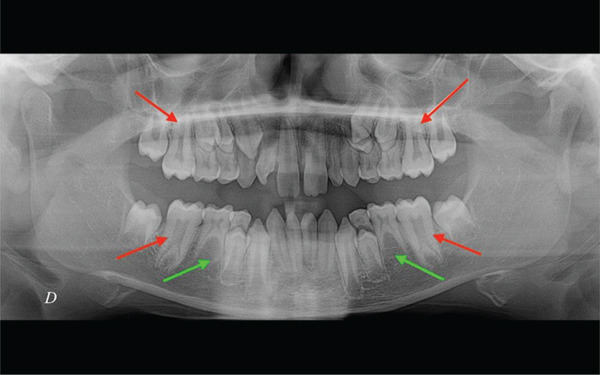
Digital orthopanomograph showing agenesis Teeth 35 and 45 (red arrow), taurodontism in Teeth 16, 26, 36, and 46 (green arrow).

Cephalometric evaluation indicated skeletal Class I with a Class III tendency and maxillary retrognathism (Figure 10).

**Figure 10:**
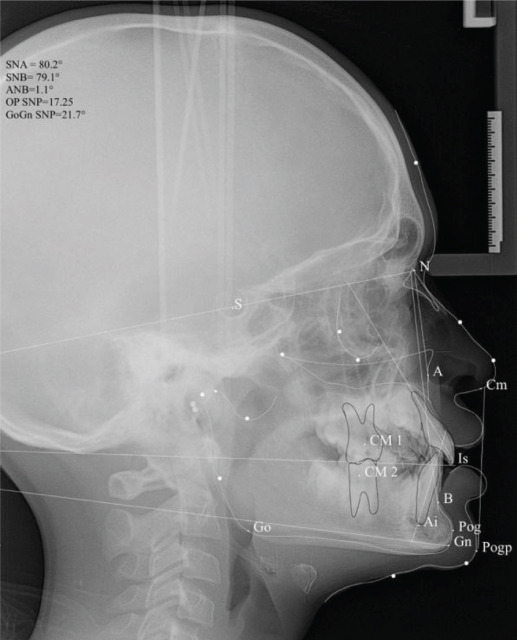
Lateral cephalometric radiograph showing a skeletal Class I pattern with a Class III tendency and maxillary retrognathism.

#### 2.4.2. Treatment Progress

Treatment consisted of direct composite reshaping using the same nanohybrid composite resin (Filtek Universal, 3 M ESPE, United States) following scaling and polishing, with an adhesive protocol identical to that described in Case 1 (Figure 11).

**Figure 11:**
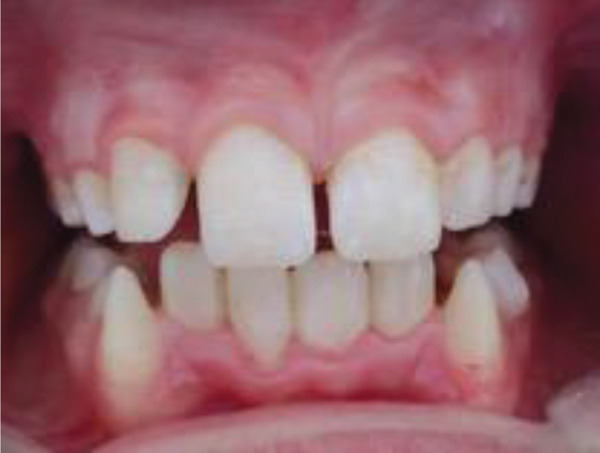
Post‐treatment photograph.

Morphological correction was achieved using an incremental layering technique. The patient was subsequently referred for comprehensive orthodontic management.

#### 2.4.3. Treatment Results

The treatment resulted in a marked improvement in dental aesthetics, accompanied by a notable enhancement in the patient′s self‐confidence and social comfort.

At the 6‐month follow‐up, the composite restorations remained clinically stable, with no evidence of fracture, marginal degradation, or discoloration. Follow‐up was conducted at 6‐month intervals over a 2‐year period, confirming the durability of the restorations and the stability of the occlusal condition.

Orthodontic treatment was successfully completed, and a comprehensive prosthetic rehabilitation plan is currently in progress to preserve arch integrity, maintain edentulous spaces, and facilitate future implant placement following completion of craniofacial growth.

## 3. Discussion

ED is characterized by considerable clinical heterogeneity, resulting in a wide spectrum of phenotypic expression even among individuals sharing a similar genetic background [4].

The present case report clearly illustrates this intrafamilial variability, as the three siblings exhibited distinct patterns of dental agenesis, tooth morphology, skeletal relationships, and psychosocial impact. This variability may be explained by differences in gene expression, incomplete penetrance, and epigenetic modulation, as well as by age‐dependent growth stages and environmental influences [4, 5, 13].

In the present cases, the severity of dental anomalies and functional impairment appeared to correlate with both developmental stage and individual adaptive capacity. The youngest patient exhibited early functional compromise requiring prosthetic rehabilitation, whereas the older siblings primarily presented aesthetic and orthodontic concerns, highlighting the dynamic nature of ED manifestations during craniofacial growth. These findings further reinforce the concept that treatment outcomes in ED are inherently heterogeneous, even within the same family, and therefore require continuous, individualized adaptation according to growth dynamics and functional demands.

Based on the family history, particularly the presence of dental agenesis in the mother and all three siblings, the inheritance pattern is suggestive of a hereditary form of ED, most likely X‐linked or autosomal dominant. However, the absence of genetic testing in this report limits the confirmation of the specific subtype and the establishment of precise genotype–phenotype correlations. It is important to note that access to molecular diagnostic testing remains limited in certain clinical settings, including Tunisia, where high costs and technical constraints may restrict comprehensive genetic investigations in routine practice. Nevertheless, this diagnostic limitation does not preclude appropriate clinical management; rather, it underscores the importance of a meticulous phenotypic assessment to guide individualized, growth‐adapted treatment strategies. Future integration of molecular diagnostics would not only confirm the specific ED subtype but also significantly enhance genetic counseling for affected families.

The observed intrafamilial phenotypic variability aligns with findings from other reported case series, where siblings with the same genetic background displayed significantly different degrees of tooth agenesis [15–17]. Some studies describe uniform patterns of oligodontia within families, whereas others report discordant presentations ranging from mild hypodontia to severe anodontia [15, 16]. Such variability is thought to reflect the complex interactions between genetic background, epigenetic regulation, and timing of ectodermal signaling disruption during embryonic development [15]. However, compared with previously reported cases, the present series highlights a broader spectrum of phenotypic divergence within a single family, emphasizing the need for individualized diagnostic and therapeutic approaches.

The dental anomalies observed in this case report, including oligodontia, peg‐shaped teeth, taurodontism, and transposition, are consistent with previous reports [6–8].

These abnormalities can significantly impair oral function, occlusal development, and facial aesthetics. Early tooth agenesis is known to impair alveolar bone development and may result in altered vertical dimension and skeletal discrepancies, as observed in the present case, where both Class II and Class III tendencies were identified [8, 10].

Recent literature emphasizes that early multidisciplinary management is essential in children with ED to optimize functional, aesthetic, and psychosocial outcomes [15, 16]. Pediatric patients often require coordinated care involving pediatric dentists, orthodontists, and prosthodontists to address complex oral and craniofacial abnormalities. In the present cases, management followed a staged and growth‐adapted approach, combining conservative restorative procedures, removable prosthetic rehabilitation, and orthodontic planning. From a clinical perspective, early intervention should be considered a dynamic and adaptive process rather than a definitive treatment phase.

Although previous studies have often reported relatively consistent patterns of dental agenesis within families, the present cases highlight that clinical presentation may be highly discordant, challenging standardized treatment protocols [17, 18].

Long‐term follow‐up is essential due to continuous craniofacial growth. In this report, follow‐up was conducted at 6‐month intervals. The two older siblings were followed over a 2‐year period, during which orthodontic treatment was completed, and prosthetic rehabilitation is currently being planned to maintain arch integrity and allow for future implant placement after growth completion. The youngest patient remains under active follow‐up to monitor craniofacial development and to periodically adapt the removable partial denture in response to growth changes. These findings highlight the importance of continuous reassessment and treatment modification over time. Importantly, early prosthetic and restorative interventions should not be considered definitive treatments, but rather transitional solutions within a long term, dynamic rehabilitation pathways.

Another important clinical consideration is the potential presence of salivary gland hypofunction in ED patients, which may lead to xerostomia and increased susceptibility to oral discomfort and plaque accumulation. This condition may negatively affect both oral hygiene maintenance and tolerance to prosthetic appliances, thereby complicating long‐term management [18].

Preventive strategies therefore play a fundamental role in the comprehensive management of children with ED. Structural dental anomalies, combined with potential salivary dysfunction, may increase susceptibility to enamel demineralization. Recent studies have suggested that remineralizing agents, including casein phosphopeptide‐based formulations and bioactive materials such as hydroxyapatite, may enhance enamel resistance and support preventive care in structurally compromised dentitions [19, 20]. Current evidence includes both in vitro findings and emerging clinical data, supporting their use as adjunctive approaches within comprehensive preventive protocols [19, 20].

The psychosocial impact of ED is an essential component of patient management, particularly during adolescence. In the present study, the oldest sibling reported significant social discomfort prior to treatment, with noticeable improvement following aesthetic rehabilitation. These findings are consistent with previous studies demonstrating that early dental intervention can positively influence self‐esteem, social interaction, and overall quality of life in affected individuals [9, 10, 21].

From a therapeutic perspective, the clinical protocols used in this study were designed to be minimally invasive and compatible with ongoing growth. Direct composite restorations were performed using adhesive systems and incremental layering techniques to optimize aesthetics and durability, whereas removable prosthetic appliances were selected to restore function and vertical dimension in younger patients. However, these approaches require periodic maintenance. Composite restorations may be subject to wear or marginal degradation, and removable prostheses require regular adjustment to accommodate craniofacial growth. These factors further emphasize the necessity of structured long‐term follow‐up.

Several limitations of this study must be acknowledged. First, the absence of genetic testing limits the ability to confirm the ED subtype and to establish genotype–phenotype correlations. Second, the small sample size restricts the generalizability of the findings. Third, although follow‐up was conducted over a 2‐year period for the older siblings, longer‐term evaluation is required to assess the stability of outcomes and the success of future implant‐based rehabilitation. Finally, outcome assessment was primarily based on clinical observations and patient‐reported improvements, without the use of standardized or validated measurement tools (such as oral health‐related quality of life [OHRQoL)] instruments) for functional and psychosocial evaluation.

Despite these limitations, this report provides clinically relevant insights into intrafamilial phenotypic variability and highlights the importance of individualized, multidisciplinary, and growth‐adapted management in pediatric patients with ED. Future studies incorporating molecular diagnostics, standardized outcome measures, and long‐term follow‐up are needed to further improve understanding of disease variability and to optimize therapeutic strategies.

## 4. Conclusion

This case report highlights the marked intrafamilial phenotypic variability of ED, demonstrating that individuals with a shared genetic background may exhibit significantly different clinical expressions and therapeutic needs. These findings emphasize that management of ED should be individualized, dynamic, and growth‐adapted, rather than protocol‐driven. Early conservative and interim rehabilitative strategies play a critical role in restoring function, improving aesthetics, and enhancing psychosocial well‐being during childhood, while preserving future treatment options. Future research integrating molecular diagnostics, standardized outcome measures, and long‐term follow‐up is essential to refine individualized treatment pathways and strengthen evidence‐based clinical decision‐making in patients with ED.

## Funding

No funding was received for this manuscript.

## Conflicts of Interest

The authors declare no conflicts of interest.

## Data Availability

The data that support the findings of this study are available from the corresponding author upon reasonable request.

## References

[1] Bergendal B. , Orodental Manifestations in Ectodermal Dysplasia-A Review, American Journal of Medical Genetics Part A. (2014) 164A, no. 10, 2465–2471, 10.1002/ajmg.a.36571, 24719393. 24719393

[2] Wright J. T. , Grange D. K. , and Fete M. , Adam M. P. , Bick S. , Mirzaa G. M. , Pagon R. A. , Wallace S. E. , and Amemiya A. , Hypohidrotic Ectodermal Dysplasia, GeneReviews, 2003, University of Washington, Seattle. 20301291

[3] Dev A. , Malhi K. , and Mahajan R. , Ectodermal Dysplasia - An Overview and Update, Indian Dermatology Online Journal. (2024) 15, no. 3, 405–414, Published 2024 Apr 23 10.4103/idoj.idoj_599_23, 38845644. 38845644 PMC11152464

[4] Ahmed H. A. , El-Kamah G. Y. , Rabie E. , Mostafa M. I. , Abouzaid M. R. , Hassib N. F. , Mehrez M. I. , Abdel-Kader M. A. , Mohsen Y. H. , Zada S. K. , and Amr K. S. , Gene Mutations of the Three Ectodysplasin Pathway Key Players (EDA, EDAR, and EDARADD) Account for More Than 60% of Egyptian Ectodermal Dysplasia: A Report of Seven Novel Mutations, Genes. (2021) 12, no. 9, Published 2021 Sep 8 10.3390/genes12091389, 34573371. PMC8468066 34573371

[5] Cluzeau C. , Hadj-Rabia S. , Jambou M. , Mansour S. , Guigue P. , Masmoudi S. , Bal E. , Chassaing N. , Vincent M. C. , Viot G. , Clauss F. , Manière M. C. , Toupenay S. , le Merrer M. , Lyonnet S. , Cormier-Daire V. , Amiel J. , Faivre L. , de Prost Y. , Munnich A. , Bonnefont J. P. , Bodemer C. , and Smahi A. , Only Four Genes (EDA1, EDAR, EDARADD, and WNT10A) Account for 90% of Hypohidrotic/Anhidrotic Ectodermal Dysplasia Cases, Human Mutation. (2011) 32, no. 1, 70–72, 10.1002/humu.21384, 20979233. 20979233

[6] Albarkheel A. I. and Odhaib M. A. , Oral Findings in Pediatric Patients With Ectodermal Dysplasia: A Systematic Review, International Journal of Applied Dental Sciences. (2023) 9, no. 3, 420–428, 10.22271/oral.2023.v9.i3f.2083.

[7] Elgasmi F. E. , Rahmaoui M. , Elarabi S. , and Badre B. , Dental Management of Ectodermal Dysplasia: A Report of Two Clinical Cases, Cureus. (2025) 17, no. 5, Published 2025 May 13e84031, 10.7759/cureus.84031, 40510078. 40510078 PMC12161414

[8] Shah S. A. Y. , Hassan W. U. , Sajjad W. , Shabbir U. B. , and Raza A. , A Rare Case of Hypohidrotic Ectodermal Dysplasia in a Seven-Year-Old Child, Cureus. (2022) 14, no. 4, Published 2022 Apr 20e24300, 10.7759/cureus.24300, 35602783. 35602783 PMC9121332

[9] Yilmaz H. , Tuncer A. V. , and Ertas G. , Interdisciplinary Approaches to Dental Rehabilitation in Hereditary Ectodermal Dysplasia, Clinical Oral Investigations. (2024) 28, no. 6, 4941–4951.

[10] Sinada N. and Wang C. I. , Fixed Prosthodontic Rehabilitation for an Adolescent Patient With Ectodermal Dysplasia Using a Fully Digital Workflow: Two-Year Follow-Up, Journal of Prosthetic Dentistry. (2024) 131, no. 6, 990–995, 10.1016/j.prosdent.2022.10.016, 36577569. 36577569

[11] Ahmadzai N. , Ehsan H. , Sapai F. , Nejabi M. B. , Khairzad A. A. , and Sharifi N. , Early Oral Rehabilitation of a Pediatric Patient With Hypohidrotic Ectodermal Dysplasia: A Case Report From Afghanistan, Clinical Case Reports. (2026) 14, no. 4, e72396, 10.1002/ccr3.72396.

[12] Nejabi M. B. , Anwari A. , Shadab H. , Mtawakel N. , Omarzad F. , and Ahmadi M. E. , Prosthodontic Management of a Patient With Ectodermal Dysplasia: Case Report, Clinical, Cosmetic and Investigational Dentistry. (2023) 15, 133–141, 10.2147/CCIDE.S419939. 37576744 PMC10416782

[13] Itin P. H. , Rationale and Background as Basis for a New Classification of the Ectodermal Dysplasias, American Journal of Medical Genetics Part A. (2009) 149A, no. 9, 1973–1976, 10.1002/ajmg.a.32739, 19353583. 19353583

[14] Riley D. S. , Barber M. S. , Kienle G. S. , Aronson J. K. , von Schoen-Angerer T. , Tugwell P. , Kiene H. , Helfand M. , Altman D. G. , Sox H. , Werthmann P. G. , Moher D. , Rison R. A. , Shamseer L. , Koch C. A. , Sun G. H. , Hanaway P. , Sudak N. L. , Kaszkin-Bettag M. , Carpenter J. E. , and Gagnier J. J. , CARE Guidelines for Case Reports: Explanation and Elaboration Document, Journal of Clinical Epidemiology. (2017) 89, 218–235, 10.1016/j.jclinepi.2017.04.026, 28529185. 28529185

[15] Morandini A. C. , Adeogun O. , Black M. , Holman E. , Collins K. , James W. , Lally L. , Fordyce A. , Dobbs R. , McDaniel E. , Putnam H. , and Milano M. , Ectodermal Dysplasia: a Narrative Review of the Clinical and Biological Aspects Relevant to Oral Health, Frontiers in Pediatrics. (2025) 13, Published 2025 Feb 271523313, 10.3389/fped.2025.1523313, 40083426. 40083426 PMC11903481

[16] Gholman R. R. , Kassar W. M. , and El Meligy O. A. , Dental Rehabilitation of a Child With Ectodermal Dysplasia: A Case Report, International Journal of Clinical Pediatric Dentistry. (2019) 12, no. 4, 362–365, 10.5005/jp-journals-10005-1651. 31866726 PMC6898874

[17] Alshegifi H. A. , Alamoudi A. M. , Alrougi A. , Alshaikh H. , Alamri A. , and Shawli A. M. , Ectodermal Dysplasia: A Case Report, Cureus. (2022) 14, no. 1, Published 2022 Jan 12e21184, 10.7759/cureus.21184, 35047314. 35047314 PMC8759711

[18] Gomes M. F. , Sichi L. G. B. , Giannasi L. C. , Amorim J. B. O. , da Rocha J. C. , Koga-Ito C. Y. , and Salgado M. A. C. , Phenotypic Features and Salivary Parameters in Patients With Ectodermal Dysplasia: Report of Three Cases, Case Reports in Dentistry. (2018) 2018, Published 2018 Mar 202409212, 10.1155/2018/2409212, 29854479. 29854479 PMC5941722

[19] Gokce A. N. P. , Kelesoglu E. , Sagır K. , and Kargul B. , Remineralization Potential of a Novel Varnish: An In Vitro Comparative Evaluation, Journal of Clinical Pediatric Dentistry. (2024) 48, no. 6, 173–180, 10.22514/jocpd.2024.137, 39543894. 39543894

[20] Scribante A. , Pascadopoli M. , Bergomi P. , Licari A. , Marseglia G. L. , Bizzi F. M. , and Butera A. , Evaluation of Two Different Remineralising Toothpastes in Children With Drug-Controlled Asthma and Allergic Rhinitis: A Randomised Clinical Trial, European Journal of Paediatric Dentistry. (2024) 25, no. 2, 137–142, 10.23804/ejpd.2024.2130, 38357755. 38357755

[21] Locker D. , Psychosocial Consequences of Dental Fear and Anxiety, Community Dentistry and Oral Epidemiology. (2003) 31, no. 2, 144–151, 10.1034/j.1600-0528.2003.00028.x. 12641596

